# “There is always a reason why someone is doing something”: The importance of life history and personhood when supporting people with dementia to “wander” in care homes

**DOI:** 10.1177/14713012251316679

**Published:** 2025-01-27

**Authors:** Yelena Mikhaylova-O’Connell, Alys Wyn Griffiths, Iria Cunha, Reena Devi, Karen Spilsbury, Mary Gemma Cherry

**Affiliations:** Department of Primary Care & Mental Health, 4591 University of Liverpool, UK; Division of Neuroscience, School of Medicine and Population Health, 7315 University of Sheffield, UK; Department of Primary Care & Mental Health, 4591 University of Liverpool, UK; School of Healthcare, 4468 University of Leeds, UK; Department of Primary Care & Mental Health, 4591 University of Liverpool, UK

**Keywords:** person-centred care, wandering, social care, cognitive impairment, positive risk-taking

## Abstract

Up to 60% of people living with dementia who reside in care homes will ‘wander’ at some point. A person-centred approach should be taken to support each person’s individual needs through tailored interventions when wandering. This study aimed to identify care home staff perspectives on what supports safe wandering for people living with dementia in care home environments. As part of a larger study, and using a person-centred framework, semi-structured qualitative interviews were conducted with staff (*N* = 19) recruited from care homes in the North of England who provide care for older people. Transcripts were analysed using framework analysis. Four themes were identified, and two of these themes are presented here. Staff highlighted the importance of ***ensuring that personhood is at the centre of care delivery*** when supporting residents to wander. Clear leadership from management and meaningful involvement of families allowed staff to provide better support for residents. Staff also reflected on the ***importance of identification of unique impacts of dementia on each individual*** when providing person-centred care. The delicate balance between safety and well-being was consistently considered and reviewed. We identified a range of individual factors that contribute towards safe and supported wandering for people living with dementia. Positive risk taking, supported by policies and procedures, such as resident safety and meaningful activity, may allow staff to manage the benefits and risks associated with wandering.

## Background

Around 60% of people living with dementia will ‘wander’ whilst residing in care homes (Jayasekara, 2009). Whilst this is arguably a fundamental human activity that we all engage in and often find enjoyable, for the purposes of this paper we define wandering as frequent and persistent walking amongst people living with dementia, that, in certain circumstances, care staff or relatives may find difficult to support (Cipriani et al., 2014; Dewing, 2006, 2011; Graham, 2017). It is important to acknowledge that language use and conceptualisation of wandering are both complex, and our own (dis)comfort with the terminology should be reflected on when supporting people living with dementia (Dewing, 2006, 2011; Graham, 2017).

Various definitions of wandering have been provided, with one focusing on observable characteristics to define something as wandering; high frequency and repetition of walking (Algase et al., 2007). Many factors, both positive and negative, have been shown to contribute towards wandering, such as increasing lifelong walking habits, severity of dementia, and boredom (Algase et al., 2007, 2007b). However, a review of terminology and definitions provided a broader consideration of wandering as movement, where the purpose and aim may not be understood by others, and bringing together physical and emotional personal needs, with environmental considerations (Cipriani et al., 2014). Furthermore, a recent review confirmed that there is no consensus on a definition of wandering (Park & Lee, 2024). Within the present paper, we use the term ‘wandering’, as this was the language choice of our participants.

Person-centred care, in which each individual’s needs, values and beliefs are prioritised, has become best practice within health and social care (Brooker & Latham, 2015; Kitwood, 1997). This encourages the integration of life history and personalisation, when designing and developing activities and providing care. Staff working in care homes are encouraged to collect information about an individual, record this, and subsequently design interventions that are personalised and integrate what is known about someone’s history (Berendonk & Caine, 2019). Previous studies have shown that people living with dementia enjoy participating in lifestory work and talking about their lives (McKeown et al., 2015). By establishing familiarity with the individual, staff are better equipped to enable the person to enjoy a greater degree of autonomy and engagement (Hedman et al., 2022). Embedded person-centred care practices result in an enhanced quality of life for residents, decreased agitation and stress, and improved working conditions for social care staff (Rutten et al., 2021). Staff should learn about the individual’s unique history, their likes and dislikes, abilities and interests, and supporting interactions with the individual with this knowledge (Rutten et al., 2021). Person-centred care is underpinned by acceptance of the person’s reality, empathetic communication, support of the individual’s interests though meaningful engagement, and creation of supporting communities which include individuals, their family members and staff (Fazio et al., 2018).

In addition to offering personalised activities, it has long been established that a person-centred approach should be taken to support each person’s individual needs through tailored interventions when wandering (Robinson et al., 2007). However, this not currently reflected within the evidence base. Most research to date has focused on implementing strategies to prevent wandering (Neubauer et al., 2018), often citing resident safety as the reason. This incorporates several concerns, such as increased risk of falls, health impacts such as dehydration and weight loss, and maintaining positive relationships between residents (Barrett et al., 2020). Systematic reviews have identified a range of strategies to prevent or reduce wandering, although no interventions are currently recommended based on the evidence base (Neubauer et al., 2018; Robinson et al., 2007; Siders et al., 2004). However, it is still unclear which strategies are successful for whom and in what context, as well as whether such strategies are trying to prevent or support wandering. Additionally, there is very little recent evidence, which may reflect the challenges of this area. One recent study asked people living with dementia why they wander and identified a range of reasons including enjoyment, purpose, lifelong habits and socialisation (Adekoya & Guse, 2019). This suggests misalignment between resident and staff perspectives on the purpose and value of wandering. There is a clear need to develop an evidence base to support staff to understand why residents wander, to encourage rather than prevent this (Backhouse et al., 2018), whilst considering the ethical and moral dilemmas presented to staff (Liddell et al., 2021).

This study aimed to explore the perspectives of staff working in care homes, on how to support safe wandering for residents. Specifically, within this paper, we aimed to explore care home staff perspectives on contribution of dementia and personhood within this, towards their role in supporting safe wandering, using a person-centred framework.

## Method

### Design

A qualitative, exploratory study was conducted, consisting of individual semi-structured interviews analysed using framework analysis (Ritchie & Spencer, 2002).

### Participants and sampling

Participants in this study were care home staff and were recruited via posters displayed in care homes in the North of England and shared via professional networks, social media, and snowballing techniques. Participants contacted the research team to express an interest in participating. To ensure participants working in various roles with a range of demographics were recruited (e.g. age, gender), purposive sampling (Palinkas et al., 2019) was used. Inclusion criteria were: anyone currently working with people living with dementia within a care home setting, as a permanent or bank member of staff. There were no inclusion criteria around specific roles or demographic characteristics, and all staff were welcome to express their interest in participating. We aimed to recruit between 16 and 20 participants in total, with a broad range of demographic characteristics to represent the social care staff population (Skills for Care, 2024).

### Procedure

A semi-structured interview topic guide informed by existing literature was developed by the research team in collaboration with experts in the social care field (i.e. care home managers, dementia care specialists) and a Lay Advisory Group consisting of relatives of someone living with dementia. The topic guide explored staff knowledge, their experiences of people wandering in care homes, and barriers and facilitators to supporting wandering at a personal and care home level. The topic guide was piloted prior to the first interview being conducted (See Appendix 1). Semi-structured interviews were conducted with care home staff between September 2022 and March 2023. Dependent on participant preference, these were either conducted in person within their workplace or via video conferencing. Interviews lasted up to 1 hour and were transcribed verbatim and anonymised. Transcripts were regularly reviewed to refine the topic guide and begin initial data analysis. Participants received a £20 voucher to acknowledge their time and contribution to the study.

### Data analysis

Transcripts were analysed using framework analysis (Ritchie & Spencer, 2002), a form of thematic analysis which allows for patterns within qualitative data to be identified, analysed and reported (Braun & Clarke, 2006). This method was chosen because it is both inductive (i.e. driven by prior knowledge and theory) and deductive (i.e. data-driven). Ritchie and Spencer’s (2002) five-step method was followed: (i) familiarisation with the dataset; (ii) framework development; (iii) line-by-line coding and indexing against the framework; (iv) charting data; and (v) mapping and interpreting patterns in data. Four researchers (AWG, MGC, IC, YM-O’C) independently familiarised themselves with the dataset and conducted line-by-line coding of two transcripts, meeting to discuss these processes and to develop the framework. Transcripts were independently coded by the same researchers, with data systematically mapped to the framework and charted. This process was repeated until coding saturation was reached. The framework was refined throughout data analysis, and consensus among researchers regarding quote placement was achieved through discussion.

### Reflexivity

We reflected on our extensive care home experience, both in practice and research. The influence of each researcher’s positionality was explored via regular discussions. This included acknowledging the impact of practice and research backgrounds in dementia care, which align with a person-centred approach. All research team members believe that wandering should be supported as a form of positive risk taking, and should not be prevented. Several members of the research team also have, or had, a relative living in a care home. Conscious attempts were made to recognise biases throughout data collection and analysis, including reflections on how to include perspectives that conflicted with the research team’s positionality, particularly around prevention of wandering. Language and terminology around wandering is complex, and within this paper, we aimed to use the language of participants throughout.

## Ethical issues

Ethical approval was obtained from {redacted} (reference 11504) prior to data collection. Participants provided written informed consent and could review their transcript prior to analysis. Participants were made aware of the potential for distress when discussing issues around supporting safe wandering, and could pause or end the interview at any point should they wish to. This was reinforced when the interview began.

## Results

A total of 19 participants were recruited (see Table 1 for an overview of participant demographics). Overall, four themes were developed. For discussion of the importance of care home environment and culture in supporting wandering, please see {redacted}.This paper presents data for two further themes that encapsulate the importance of considering personhood and life history, and understanding the unique ways that dementia affects each resident when providing person-centred care (see Table 2). Themes and sub-themes are presented here as headings and sub-headings.

**Table 1.** Participant demographics.

Characteristics	*n*
Age	
20–34	5
35–49	9
50–64	5
Gender	
Female	16
Male	3
Job role	
Manager/Deputy manager	10
Care staff	5
Activities coordinator	1
Registered nurse	3
Geographical location	
Leeds	11
Manchester	1
Liverpool	4
Norwich	1
York	2
Ethnicity	
White British	15
Other white background	2
Black African	1
Asian British	1

**Table 2.** Summary of main and sub-themes.

Main theme	Sub themes
Ensuring that **personhood is at the centre of care delivery**	Seeing **wandering as a part of daily life** for residents
	Having **meaningful involvement of families** in supporting residents
	**Clear leadership from the manager** around strategies to support wandering
	**Personalisation of strategies to support wandering**
**Identification of unique impacts of dementia** on each individual	The **interplay between nutrition, hydration and weight** for residents
	Understanding **why is someone wandering?**
	**Balancing safety with well-being** to support people to wander safely

## Theme 1: Personhood at the centre of care delivery

When providing person-centred care, all staff spoke about the importance of considering each individual’s life history and how walking or wandering had been embedded in their daily life. Working closely with family members, to help alleviate concerns about wandering and reframe this as a positive aspect of care helped to ensure that residents felt supported to wander. Clear leadership from management helped staff to feel confident in supporting residents to wander, developing and refining personalised strategies to support each individual.

### Influence of life history

Recognition that residents have lived fulfilling lives at the point that they entered the care home allowed staff to consider ways that their individual life history may influence their present behaviour. Residents’ previous careers were seen as potential influences on their decision-making, particularly regarding waking times and activity levels throughout the day. Drawing together someone’s life history and their current preferences and behaviour helped staff to support individuals to wander.

“If someone’s been a milkman or a window cleaner all their life, they may get up at 6:00 in the morning and still have that drive to get up. So, it’s understanding the story behind {wandering}.” (ID15)

Staff reflected that residents have different energy levels and expectations regarding activity. Those residents that had lots of energy, which could not be burned through usual care home routines, and those that have always had active hobbies were more likely to spend time wandering.

“She used to do a lot of physical exercise. She used to go fell walking. She’s not a frail woman. She used to do line dancing. She probably has a lot of energy that is not being used up, therefore to wander, it actually kills a bit of energy and then she settles.” (ID18)

Participants worried about boredom as a contributing factor to wandering, reflecting that the sudden change of pace within the care home compared to the pace residents had set for themselves prior to moving into the care home, could be challenging for some and result in wandering as a means of occupying time.

“I think she’s just looking for stuff to do because she’s always been a busy woman and she’s always been on her feet.” (ID08)

One participant reflected that they felt that all residents should be encouraged to wander, as without this, they had minimal variation in their environment or company throughout the day as compared to a time before they lived in a care home setting.

“I feel like if they don’t {wander} then it becomes very restrictive for them.” (ID05)

### Wandering as a part of daily life

Understanding how walking or wandering had played a part in residents’ lives to date helped staff to understand their choices, particularly for those residents who were used to daily exercise. Participants were keen to facilitate this where possible, but acknowledged the need to learn about each resident’s preferences and routines to do this effectively.

“They might just want a bit of exercise. They’re used to walking, they want to keep up with the exercise. It’s individual really. It’s just finding out how we can support them.” (ID12)

Without the opportunity to exercise, residents may struggle to sleep at night, which impacts their routines.

“Some of them wander purely as an exercise, just to get a bit of walking done for the day. And actually, it’s quite helpful because then they get tired naturally and then go to bed. But sometimes if they don’t do that then they can stay up until the middle of the night, which is not good because it messes up their patterns.” (ID06)

Participants reflected on the importance of not seeing residents as people who had *previously* been active, but people who remain active whilst restricted by the environment.

“Our residents are used to being very active people and they still are active people even though they’re living in the care home. So, it’s about knowing the person and if they are not distressed and they are quite content it’s fine.” (ID07)

Safety concerns, particularly around falling, were named as the only reason to stop someone from wandering. The importance of not telling someone to sit down was mentioned.

“The odd time we say can you sit here to drink your coffee, but we’ve not to tell people to sit down unless there is a reason why. They might be unsteady on their feet. But as a rule, no, they can wander about wherever.” (ID11)

Participants reflected on how they would want their own relatives to be supported, drawing comparisons that allowed them to be empathetic to residents’ situations. They outlined the potential ‘knock-on effects’ from preventing someone from wandering, in terms of their physical and mental health.

“Personally, if my granddad was here, who has dementia, he would walk because his daily routine was going for a walk around the park. I wouldn’t want to stop that because we would reduce his mobility, reduce his hunger, reduce his stimulation. It all would have a knock-on effect. Whereas if we just allow him to have that morning walk to keep to his routine, keep him feeling hungry, less bored, keep up with his mobility, reduce his falls, it just has such a positive effect on him and it would have a positive effect on other people as well.” (ID12)

Participants also acknowledged that people living with dementia are human, and are likely to express a range of emotions related to their environment, such as frustration or a desire to leave.

“In the same way you or I may think, “I can’t wait to get out this house.” That can also be a thing, just the natural human thing of I’m fed up, I’m tired, I want to get out of here.” (ID18)

For others, lifelong enjoyment of being outdoors and exploring the countryside could lead to wandering, which staff were happy to support. In doing so, staff spoke to the resident about what they were doing, and encouraged them to continue.

“I’ve met a gentleman previously and because he did a lot of walking out in the countryside, it was very apparent that that's what he felt he was doing.” (ID01)

Striving to provide person-centred care could also lead to inequalities in access to time outside the care home. In one case, a participant reflected that only those who wander are offered an accompanied walk in the park. Participants saw this as a “*change from just wandering to having a helpful walk*”. This shift in language is interesting, as residents spend the majority of their time walking within the care home.

“There are a few who do like to wander, and we’ll take them to the local park. They really enjoy it and you can really see they’re stimulated and responsive to their surroundings” (ID16)

However, staff acknowledged that not all residents enjoyed wandering. This highlighted the importance of person-centred strategies but became exasperated when strategies did not work. Participants reflected on these situations with fondness and humour.

“With one resident I said, “Right, it’s a beautiful autumn day. Get her out for a walk.” And she was not happy at all. She came back and she was, like, “I’m freezing, and I did not enjoy that.” You know, when you just, kind of, go, “You can’t please all of the people all of the time.”” (ID01)

### Meaningful involvement of families

Participants viewed families as integral to supporting wandering within care homes. Participants encouraged residents to walk with their families, seeing it as a meaningful activity enjoyed together. They perceived that relatives “*embraced*” wandering and would spend time walking on paths, and around corridors with their relative. This was particularly noticed in good weather, where outdoor spaces were available. This was considered as families supporting staff, providing residents with some dedicated time. Walking together could be seen as a therapeutic interaction, giving the opportunity for reminiscence, and providing comfort to the resident through touch and reminiscence.

“A lady yesterday, her family came to visit, and it was a lovely therapeutic interaction between them. They just held her hand. She loves having her hand held. And they just walked together around the home. And as they were doing it, they were reminiscing about travelling up to Scotland and climbing up the hills.” (ID01)

For some, this involved leaving the care home with family members, which provided residents with stimulation and a change of environment.

“{Residents} will be taken out by their relatives, and that’s really good, really stimulating for them. They can take them out to the park, or out for coffee.” (ID06)

Participants saw residents wandering as demonstrating delivery of person-centred care, and felt positive when communicating with families that their relative was wandering when they visited.

“It’s nice when they come because the resident’s family member will say, “Oh, where’s so and so?” And it’s, like, “Ah, I think they’re down there…”” (ID11)

Sometimes families held a conflicting perspective on wandering, which was difficult to manage. This required transparency between staff and relatives to develop trusting relationships that allowed the person to wander safely, whilst acknowledging concerns around safety. Relatives were particularly concerned about their loved one falling or leaving the care home without appropriate supervision or support. Concerns were also raised about quality of care, with specific regard to nutrition and hydration.

“Family members do tend to worry about wandering, because they worry that they’ll be able to get out, that you’re going to lose track of what they’re drinking, that it’s a lack of care from staff.” (ID18)

Participants reflected that for some families, the resident may have moved from their own home, to hospital, to the care home in quick succession. They acknowledged the importance of time in helping families understand and accept how wandering can be supported. This required staff to share this information at a pace that was appropriate for the relative, without overwhelming them.

“So it just takes time for them to understand what the routine is here because being at home, being in hospital and then in a care home is all three different environments.” (ID05)

Participants saw a role for themselves in educating families about how safe wandering is and should be supported. One participant used a monthly newsletter to help share information, whereas other participants spoke to families on a one-to-one basis.

“Sometimes with families they are having a hard time to understand why their mum, dad, uncle, aunty, is doing this, so we talk them through it.” (ID02)

Families were seen as integral in developing strategies to support wandering together, allowing staff and relatives to learn from each other. Working together through information-sharing and creating care plans was seen as an opportunity to strengthen relationships.

“It’s educating the families as well as sitting with them and trying to work it out together, working alongside the family, saying, “Well, it could be this,” or, “Shall we try this?” And working together to find a solution. I find that that bonds us as well with the family.” (ID15)

Participants viewed relationship-building as working best when it began as soon as the resident moved into the home. The unique knowledge that a family hold about a person from before they have dementia was integral to developing appropriate strategies, for example, knowledge about their preferences, likes and dislikes.

“That relationship between the home and the families are so crucial because when you work together, you can build a productive plan. Also complete transparency between both.” (ID18)

This was particularly important where a positive risk taking approach was taken. Not all families were receptive to this with some displaying fear or anxiety, particularly around the likelihood of the resident falling, and whether wandering could contribute towards this. Participants saw value in investing time and effort in explaning their approach to families to get everyone “*on the same page*”. Participants reflected that resident safety was at the heart of all concerns.

“If families say, “My loved one keeps falling, why are you not stopping…?” It’s about that education, it's, “We can’t stop people falling. We can’t restrict because that’s going to have a negative effect as well. All we can do is reduce the impact of risk.” And we can show the evidence, the success stories of doing that and the impact that it has on the residents. Sometimes it takes a while for the family to come round to that and understand that.” (ID15)

Participants reflected on the importance of families having confidence that staff are able to support their relative to wander safely. This was seen as a staff responsibility, with transparency and openness with families key to success. Where families have less knowledge about dementia, particularly how symptoms may fluctuate over time, staff saw a role for themselves in working together to help relatives develop confidence.

“It’s our job to make them feel that they’re being properly looked after and, if they are wandering, that they’ve got support. I think as long as they’re confident that we know their needs and we support them in as many ways we can, then they’re okay.” (ID06)

At times, relatives were able to encourage staff that they were doing the right thing, when staff worried about a resident. Relatives could advocate for the resident’s preferences through co-creating a care plan to support them, which could be regularly updated.

“There’s one lady who will walk all day with the odd little rest to have a sleep. Before, we used to try and get her to sit down to rest her legs, but her husband was like, “No, just let her walk around. She will eventually sit down when she’s tired,” but sometimes she can go about four or five hours walking up and down the corridors non-stop, but like he says, let her do it.” (ID14)

The relationships between residents who become each other’s chosen friends and family within the care home was also considered, as people may wander to spend time together and enjoy each other’s company. This was seen as a valuable way to spend time, which should be encouraged.

“The relationships with the other residents with dementia, they’re really strong, aren’t they?” (ID10)

### Clear leadership from the manager

As with many aspects of care, clear leadership from the manager helped staff to feel confident supporting residents to wander. Managers were seen as experts in dementia care, with extensive experience that staff could learn from. A wandering policy, with a directive approach for how to support this, was well received. Participants benefitted from collaborative working relationships and a collective response to challenges, which provided the freedom to support residents.

“We have clear guidance that all residents should have the independence to go where they want to, but that we should make sure that they are safe.” (ID05)

Creating and fostering a sense of community, through encouraging residents to move around both inside and outside the home and develop relationships with others, provided an environment where residents felt secure to wander.

“It’s just a home where people can make friendships and see other people on other floors and mix and integrate. Yes, it’s just, hustle bustle busy which is really nice.” (ID10)

Leadership was demonstrated through documentation of supporting wandering within care plans and ensuring regular updates and audits. Managers encouraged staff to learn from each other, through practice-based learning and communication around wandering.

“We have care plans for each resident. So that’s constantly assessed and updated, and if there is an issue with it, then they will look into ways to, sort of, help that. I think she’s very supportive of anything that’s going to improve the residents’ life at the care home.” (ID06)

However, within other care homes, wandering was not incorporated into care plans. Some participants reflected that this would be an important area to capture, and suggested practice changes to address this. The value of capturing this within care plans was recognised.

“I’m going to be honest here with you – none of us put it in. You’ve just raised that and I’m thinking, “I’ve never put that in. I’ve never actually written that down and said, ‘So and so likes to walk about, likes to go on into people’s rooms.’” So maybe it’s something we should look at it.” (ID09)

This was felt to be an oversight, and misaligned with other similar areas of care. Direction from the manager around what areas of care should be focused on, highlighted the importance of supporting wandering, however this was not necessarily documented anywhere.

“Nobody ever says, “We should be promoting personal care. We should be promoting laundry.” Do you see what I mean? It’s something we seem to be missing out on” (ID08)

### Personalisation of strategies to support wandering

Participants reflected on potential strategies that could be implemented based on someone’s life history, striving to avoid anything that would patronise residents, whilst recognising the importance of supporting individuals to have a sense of purpose.

“We haven’t got any prams or anything for people to push. It might be a good thing to do, to actually have prams for some of our ladies who move about, who might want a purpose.” (ID03)

Wandering was seen as something that people living with dementia can choose to do, within their own home. Participants did not feel that staff should be preventing this, as the person should have control over how to spend their time. The focus was ensuring on each resident was happy and safe.

“This is their home and they should be allowed to do what they want to do.” (ID12)

This was also considered in relation to residents feeling safe and able to express themselves. This important distinction of who controlled the environment helped staff to deliver person-centred care. Psychologically, it was felt that residents were aiming to be somewhere that they felt comfortable.

“In a nursing home, we always say, “I work in that person’s home. They don’t live in my workplace. There is that acknowledgement that you have to be aware that you are in their home and they have the right to feel safe.” (ID18)

Participants reflected that changes in wandering behaviour could be an indication that something has changed health-wise for the person. Noticing this alerted staff to involve external healthcare professionals, who could assess any changes in their mental or physical health.

“If someone who usually walks stopped walking or somebody who doesn’t walk is trying to walk more, that usually indicates that something's changed in them, and that’s when you start investigating and thinking, “Right, let’s look at all the physical things, is this a mental health thing?”” (ID15)

Being able to gather information about a resident’s life history, interpret this, and use this knowledge to implement strategies is a complex skill that requires reflection, adaptation and resilience.

“It’s very person by person, but ultimately it's about having that skill to know your resident, know the life history and that interpretation. It takes very skilful staff members to be able to understand and then implement a response to it.” (ID15)

In summary, this theme highlighted that there are many underlying reasons why someone may be wandering, and seeing each resident as a unique individual with their own history, helped staff to understand and support someone to wander safely. Challenges arose where families were concerned about increased risk of falls, or where the person became upset or distressed about their location.

## Theme 2: Identification of unique impacts of dementia

Participants highlighted that dementia affects each individual differently, and learning about residents helped to improve the quality of care they received. With wandering, this allowed staff to know when to implement specific strategies to support the individual, or when to step in and guide someone to a different activity. Identification of unmet needs helped to distinguish between wandering that staff would want to support, and wandering to seek relief from an unmet need.

### Why is someone wandering?

Dementia impacted on wandering in a range of ways, through comfort seeking, confusion around location or changing habits. Staff felt their role was to support residents to wander safely, to reduce any distress or anxiety, and to encourage residents to enjoy exploring the care home environment. Although, at times, wandering could be related to an unmet need, such as needing to go to the toilet or seeking comfort from someone. Two forms of frustration were observed; firstly, frustration with the environment which could lead to an individual wandering. Secondly, frustration with communication with staff, where a resident may become frustrated by not being able to communicate their reasons for wandering, which could in turn exacerbate the frustration they were feeling.

“Wandering can be trying to look for a familiar face for example a staff member.” (ID18)

The impact of dementia on verbal communication meant residents were not always able to communicate their reasons for wandering with staff.

“Having the time to be able to spend with that resident on a one-to-one basis to understand why they’re wandering, if there’s any emotion behind it. That takes time because with dementia you're not going to get a straight answer.” (ID05)

Conversely, for others, frustration was thought to underlie some people wandering, particularly for those who were unable to communicate this verbally. Developing relationships that helped staff to use body language as an additional form of communication helped to reduce residents’ frustration where they struggled to communicate their needs verbally.

“I think sometimes it’s the resident’s frustration. But because of obviously the diagnosis of dementia, they can't express that, can they? They can't tell you, “Listen, I'm just fed up. I want to get out,” and maybe {you should} spend time talking to them or something like that.” (ID17)

For others, wandering was seen as a form of communication of their needs or wishes, particularly for those who may struggle to communicate verbally. These may be urgent needs, or more general preferences. Staff learned over time how to use the locations that residents wandered as a way of understanding unmet care needs.

“There is always a reason why somebody is doing something. If you can begin with understanding that people with dementia have a purpose still and they’re doing it because there is a reason, you try and understand what that is. If they’re trying to pick a book off the lift door you guide them to where the books are, it’s their way of communicating with you.” (ID10)

Participants identified residents who regularly wandered to specific locations, and for whom wandering was thought to be an enjoyable activity. It was thought that residents may “*struggle to process their thoughts and feelings*”, which could lead to them wandering as a response to these challenges. Participants felt that for some people, wandering was a way to destress or decompress within the care home environment.

“We’ve got three or four people that are generally regularly on the move. In the dementia community, you'll generally see similar people rotating around the building…” (ID15)

Importantly, participants reflected on what was lost with a dementia diagnosis. Many residents were not used to being sedentary, therefore engaged in wandering to keep active. This comparison enabled staff to empathise with residents’ desire to wander.

“We’re all so busy all the time and then dementia takes the busy away, doesn’t it?” (ID03)

Participants reflected on the importance of someone wearing their own glasses and having regular sight and hearing tests. Residents may not be able to communicate a sensory impairment, or discomfort. Supportive, well-fitting clothing and footwear was also recommended by participants. However, this required participants to spend time with the resident checking that items were appropriate subject to fluctuations in their weight and swelling in their feet, time which was not always available.

“We do encourage suitable, practical footwear that’s supportive.” (ID10)

### Interplay between hydration, nutrition and weight

Participants looked for opportunities to increase nutrition and hydration. Concerns were raised around weight loss, and the associated increased likelihood of falls, for participants who wandered regularly. Strategies were put in place to ensure that all residents had access to drinks whilst wandering, such as making meal shakes that residents could pick up and walk around with. One care home had a corridor-based “hydration station”, where drinks and snacks were always available.

“The main thing is to make sure they’ve got the right nutrition and drinks for them as well. So, we have a hydration station, so they always have access to drink and food if they want.” (ID07)

Participants reflected on the increased challenge of providing adequate nutrition and hydration for residents who wandered frequently. Being responsive to residents’ location helped to increase their access to drinks and snacks.

“If people are wandering in and out between the lounge and the room, I’ll leave one cup of coffee in the lounge, one cup of coffee in the room so I know that whatever environment they’re in there’s a drink.” (ID18)

The importance of weight monitoring, with regular documentation, enabled strategies to be adapted in response to any changes in residents’ behaviours.

“The challenge for me is weight loss that comes with constant exercise because one of my ladies who walks incessantly, she eats like a horse. But she’s losing weight because she just does not stop moving at all. She never stops.” (ID03)

Participants were concerned about residents who were unable to put weight on as they were “*always walking or dancing*”. Concerns raised about weight loss were escalated to the manager, and strategies to address this included offering food and drink outside of mealtimes, and learning resident snack preferences. Staff were required to be responsive to changing preferences.

“So it is important to get to know them, that this is what they like, get the food and drinks into them when you can…” (ID02)

### Balancing safety with mental well-being

Participants reflected on the need to balance resident safety with well-being and enjoyment of living in a care home. The onus was placed on staff to understand why someone was wandering, and to formulate a strategy to support this. Participants felt that residents could communicate when they were bored of wandering and wanted to do something else.

“You can walk with them. You can find out if there’s a need, are they hungry, do they need the toilet, are they bored? Do they just want exercise and then you can walk, you can talk with them. Do they just need distraction? There’s always a reason why somebody’s walking.” (ID12)

Participants discussed how walking with a resident could reduce the resident’s anxiety. The importance of physical touch and comfort to alleviate anxiety were highlighted. This could be seen as a “*therapeutic intervention or reassurance*” to avoid increasing someone’s distress.

“There have been times where I’ve found myself walking with residents. We used to have a lady and she would walk up and down, up and down. She suffered really badly with her anxiety, so I found myself with her, holding her hand and walking up and down the corridor.” (ID09)

Anxiety was particularly heightened in situations when it was felt that a resident was unfamiliar within the environment, possibly due to confusion related to dementia. Considering this alongside boredom from spending time in the same place, wandering could ensure that emotional needs are met. Reflection on the impact of not supporting someone to wander, and how this could affect their well-being on a longer term, was also considered.

“Because I think the difficulty with somebody that's becoming quite distressed, once you've got all that adrenaline pumping through the body, it’s really hard then to settle back down.” (ID01)

One strategy frequently mentioned was sensor mats. These could be used to support or prevent wandering. Alerting staff that someone had moved could help them to support them to mobilise, reducing the risk of them falling before they had their mobility aid.

“If you see they’re at high risk of falls, you get a sensor mat for them so that tells you they are about to get up, because you can’t really stop them, but at you’re there to actually see them as they mobilise.” (ID13)

In summary, this theme highlighted a range of ways in which dementia impacted on someone’s wandering. Challenges presented for staff in terms of understanding and responding the unique reasons underlying each resident’s wandering. Participants were able consider wandering from the perspective of someone living with dementia, to help understand how dementia may encourage someone to wander, whilst also impacting on communication. Ensuring that residents received sufficient food and drink to provide them with energy for wandering, and without causing weight loss, was a priority for staff. Specific strategies were put in place to encourage hydration and nutrition. Staff were required to maintain the delicate balance between resident well-being and enjoyment of wandering, with potential increased risk of falls and concerns about their safety.

## Discussion

The present study aimed to explore staff’s perspectives on how individual factors affect wandering within care homes, specifically considering how this helps them to support safe wandering. Staff currently have limited guidance on how to support residents who wander to do so safely. Here, we presented two overarching themes related to the importance of considering each person living with dementia as an individual.

Participants discussed how important personhood of each resident is to the care they deliver. In line with existing evidence, participants felt that residents benefit from wandering and thus retaining autonomy over their location and choice of activity (Robinson et al., 2007). Another important benefit highlighted by participants was its positive impact on the physical and mental health of residents. In particular, care staff recognised that some residents engaged in wandering to alleviate the feelings of anxiety and restlessness, and that supporting wandering meant helping residents to maintain their mental health. Previous studies explored walking in relation to mental health benefits, from the point of view of care home residents living with dementia (Adekoya & Guse, 2019). Further research to understand on staff perspectives on the impact of wandering on residents’ mental wellbeing should be considered.

Increased recognition of the benefits of wandering can help foster a culture of supporting wandering (Gu, 2015). Participants valued clarity in managers’ approaches to supporting residents to wander, with some emphasising the importance of having a clear policy on wandering. Whilst most felt their approach to positive risk taking was shared by their colleagues and senior management, some were unsure how to implement this consistently in their daily care practices. These findings are in line with existing evidence from previous care home studies which demonstrated an association between clear leadership and successful implementation of person-centred care practices (Moenke et al., 2023; Rutten et al., 2021).

The unique ways that dementia impacts each individual were considered. Staff sought to understand the circumstances which led to someone wandering, which requires high levels of knowledge and skills. Our earlier paper highlighted the importance of culture when supporting wandering (Griffiths et al., 2024). Drawing together their knowledge, with the freedom to take a positive risk-taking approach, leads to the implementation of individualised strategies drawing together someone’s life history and current preferences, which is thought to reduce the risks associated with wandering (Backhouse et al., 2018). Staff training to develop this initial knowledge is crucial and should be prioritised (Barrett et al., 2020; Griffiths et al., 2024). However, staff also benefit from clear policies and procedures to follow, including risk assessments and dedicated time to supporting people to wander safely (Barrett et al., 2020).

In the present study, participants highlighted the role that relatives play in supporting wandering, either by walking with their relative, or by helping staff to understand their relative’s life history. To date, this remains under explored within the existing literature, and future research should integrate the perspective of relatives. This could be achieved through qualitative methods such as walking interviews, or ethnography incorporating observations, where researchers could observe dyads walking together and ask about their experiences.

Whilst the topic guide specifically asked about staff support of residents when wandering, participants discussed situations where a resident may be confused or disorientated, where they may not support wandering. Trying to disentangle this is complex, and requires more in-depth work with care homes, over a prolonged period, to establish how individuals are supported to wander safely, and in which circumstances this is not possible. This also provides an important observation on current perceptions of wandering in care homes, where individuals may experience differing levels of comfort with positive risk taking. This is in line with evidence that questionable practices can arise despite good intentions, due to concerns about risks and impacts of behaviours such as wandering (Backhouse et al., 2018).

Language around wandering remains challenging and stigmatised within care homes. Participants used a range of terms, such as “exploring the environment”, to describe wandering behaviours. However, we also noted an interesting shift in language when participants discussed walking in the care home, compared to in other environments. Walking within the care home was consistently described as wandering, whereas the same participants described a ”walk in the park” or “going out for a walk”. This is particularly pertinent where the reason for wandering is seen to be a reduction of distress, or an expression of frustration, rather than for enjoyment. Such findings warrant further investigation to understand how and why this distinction is drawn, and what impact this may have on attitudes towards wandering.

### Strengths and limitations

The present study aimed to understand perspectives on wandering in care homes. This study provided an in-depth exploration of the views of care home staff working in a range of roles, around how to support residents when wandering in care homes, which to date has not been explored. Participants were recruited from a range of care homes, in the North of England. To increase inclusivity, participants chose whether to participate face-to-face or remotely (via call or video call). The work offers novel insights in this field, contributing towards addressing important knowledge gap: (1) developing guidance to support staff to reflect on why someone living with dementia might wander, and (2) how to provide person-centred care to these individuals (Backhouse et al., 2018). It also significantly contributes to the ongoing discourse about the most appropriate language to use: study participants chose (unprompted) to use the word wandering, when asked to share their preferred term. Although a contested term in published research, it is clear from this study that *wandering* is a term used within social care settings. This is important when considering how to approach this topic for practice or research. Importantly, the present findings can be applied to other settings where people living with dementia receive care and may wish to explore the environment, such as hospitals and hospices. Sharing knowledge of how to safely support wandering should improve well-being for people living with dementia, regardless of their setting.

There are several limitations within the present study. The sampling strategy encouraged participants to self-select, and therefore those who were interviewed may have particularly positive attitudes towards wandering in care homes. This is particularly pertinent as over half of participants were in leadership roles, and therefore the strategies that they identified may not be in place within all care homes, despite their recommendations. Furthermore, participants were recruited from research-active care homes, who engage with care home research networks. Therefore, the views of participants may not reflect the wider social care sector. Due to the exploratory nature of this work, and the stigma around wandering in care homes, trust between researchers and participants was particularly important, which may be reflected in those individuals who chose to take part.

### Clinical implications

The clinical implications of supporting wandering are discussed in our earlier paper (Griffiths et al., 2024). However, specifically related to personhood, several pertinent points should be considered. Care home residents have described a sense of purpose and enjoyment when wandering, conflicting much of the existing literature which relates wandering to agitation or distress (Adekoya & Guse, 2019). Routine implementation of individualised non-pharmacological interventions should be implemented, to reduce the risks associated with wandering and encourage staff confidence in supporting wandering (Backhouse et al., 2018). Regular ongoing assessment and review is required to ensure that the strategies implemented are appropriate and enjoyable for each individual (Barrett et al., 2020), acknowledging how preferences may change over time as dementia progresses.

This research was conducted within a social care sector where there are increasing staff shortages, high levels of turnover, and recent governmental policies (e.g. changes to UK immigration policy) that are likely to exacerbate these issues. Any strategies put in place to support wandering must consider these issues, for example, embedding practices into existing daily care home routines. This might include low-cost strategies such as incorporating wandering into care plan reviews or focusing on wandering within staff handovers. Strategies must also be co-designed with care home staff, residents and relatives, to increase likelihood of implementation.

## Conclusions

The present study identified the importance of considering the individual life history and personhood of people living with dementia when developing strategies to support wandering. Positive risk taking is required to ensure that staff have the confidence to balance the benefits and risks associated with wandering, acknowledging the importance of resident safety, whilst also considering the importance of meaningful activity. Clear leadership from management improves staff confidence to support residents to wander. Future research should draw together the perspectives of residents, relatives and staff, to provide a greater understanding of how life history and personhood interact with wandering in care homes.

## Appendix 1. Interview Topic Guide

1. Demographics and background

 • How long have you worked in care homes?
 • When did you start working with people affected by dementia?
 • What drew you to working with people affected by dementia?

2. Knowledge about wandering for people with dementia

 • What is your understanding about why people with dementia may wander?
 • Where do you get your knowledge about this? How do you keep this up to date?
 • Do you think people with dementia should wander in a care home setting?

3. Experience of supporting people with dementia to wander

 • What are your experiences of people affected by dementia wandering in the care home?
 • Do you support people with wandering in your role? What kind of things do you do?
 • What stops you from supporting people to wander?
 • What helps you to support people to wander?
 • How do you contribute to the design of the care home environment?

4. Care home wide initiatives

 • What strategies are in place to help people wander?
 • What strategies are in place to stop people from wandering?
 • What do you think relatives of residents think about wandering?
 • What do you think your manager thinks about wandering?
 • What do you think your colleagues think about wandering?
 • Do you think your care home is a safe place for people to wander?
 • How could the environment be changed and what impact would this have for people wandering?

5. Education and training

 • Was wandering related content included in your training?
 • What do you think are the most important things you need to know to support people wandering?
